# Amiodarone Induced Thyrotoxicosis and Treatment Complications in a Man With Cyanotic Congenital Heart Disease: A Case Report

**DOI:** 10.3389/fcvm.2020.574391

**Published:** 2020-10-30

**Authors:** Marvin Wei Jie Chua, Shao Feng Mok

**Affiliations:** ^1^Department of General Medicine, Sengkang General Hospital, Singapore, Singapore; ^2^Division of Endocrinology, Department of Medicine, National University Health System, Singapore, Singapore

**Keywords:** amiodarone induced thyrotoxicosis, treatment, complications, cyanotic congenital heart disease, thyrotoxicosis

## Abstract

**Background and Case:** Amiodarone induced thyrotoxicosis (AIT) is a potentially life-threatening condition that exists in two main subtypes – AIT Type 1 (AIT1) and AIT Type 2 (AIT2). AIT1 is a form of iodine-induced hyperthyroidism with increased thyroid hormone synthesis, while AIT2 is a form of destructive thyroiditis with increased release of pre-formed thyroid hormone. This case report describes a patient with cyanotic congenital heart disease, who developed AIT with severe biochemical thyrotoxicosis. Due to complications to corticosteroids and thionamides, second-line treatment with cholestyramine and lithium was given which eventually restored euthyroidism, averting the need for thyroidectomy and its associated risks. Due to the presence of both typical and unusual features, the final diagnosis of AIT2 could only be retrospectively elucidated after a prolonged clinical course.

**Conclusion:** Corticosteroids are well-recognized to be the first-line treatment for AIT2. This case illustrates a rare phenomenon: successful treatment of AIT2 with lithium and cholestyramine. In patients who develop complications from first-line therapy, prompt treatment with alternative agents may successfully avert thyroidectomy and its associated risks.

## Introduction

**Figure 1:**
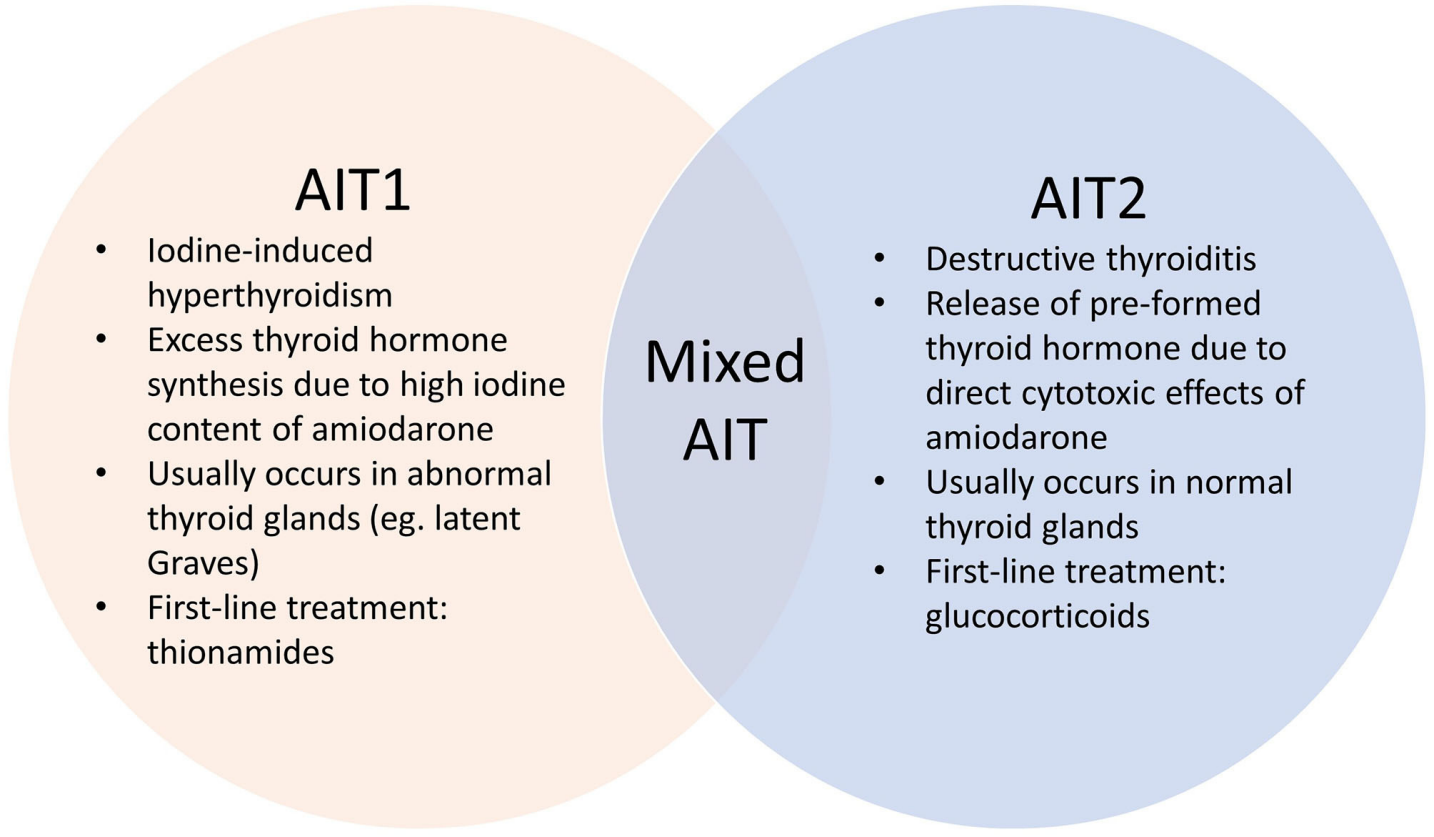
Pathophysiology of various AIT subtypes.

Amiodarone induced thyrotoxicosis (AIT) is a potentially catastrophic situation for patients with cardiac disease (1) who are at risk of life-threatening complications. AIT exists in two main subtypes – AIT1 and AIT2, which have different underlying pathophysiological mechanisms (Figure 1). AIT1 is a form of iodine-induced hyperthyroidism caused by the high iodine content of amiodarone, while AIT2 is a form of destructive thyroiditis caused by direct cytotoxic effects of amiodarone and its metabolites.

Diagnosis is often delayed and differentiating between AIT1 and AIT2 is often difficult.

In this case report, we describe the prolonged and tumultuous journey of a young man with cyanotic congenital heart disease who developed AIT. In our bid to urgently reduce serum thyroxine to prevent cardiac decompensation, we encountered significant barriers – the development of complications to first-line therapy, which necessitated the use of second-line agents. It did not help matters that the AIT subtype remained unclear throughout this entire process. Eventually, we managed to successfully restore euthyroidism with lithium and cholestyramine. The successful use of lithium and cholestyramine in the treatment of AIT, let alone AIT2, is rarely reported.

## Case Presentation

The patient was a 32-year-old gentleman with complex cyanotic congenital cardiac disease consisting of total anomalous venous drainage, double outlet right ventricle and severe pulmonary stenosis, leading to a uni-ventricular circulatory system (ejection fraction was 45% in April 2017). This was complicated by atrial flutter and complete heart block, which were treated with radiofrequency ablation and permanent pacemaker insertion. Long-term medications included amiodarone which he was taking for the last 3 years due to the recurrence of atrial flutter post-ablation (100 mg daily), digoxin (62.5 mcg daily), bisoprolol and warfarin.

The clinical course described spanned 8 months (January to August 2017). Thyroid function test (TFT) monitoring was performed due to long-term amiodarone use. Primary hyperthyroidism was first diagnosed in January 2017, with free thyroxine (fT4) > 72 (reference interval 8.0–16.0) pmol/L and TSH <0.01 (reference interval 0.45–4.50) mIU/L (Table 1), during an admission for lower limb haematoma from warfarin over-anticoagulation. The latter was not a co-incidence, as thyrotoxicosis is known to potentiate the effects of warfarin by increasing the degradation of coagulation factors (2). In addition, pharmacokinetic interaction between amiodarone and warfarin leads to decrease in warfarin requirements, although in our patient amiodarone therapy was long-standing with no changes in dosage (2).

**Table 1:** TFT Trend while patient was on amiodarone.

**Investigations**	**Reference interval**	**Number of months patient was on amiodarone**				
		**0**	**3**	**9**	**29**	**32**
fT4 (pmol/L)	8.0–16.0	13.6	**19.2**	**20.4**	**26.0**	**>** **72.0**
TSH (mIU/L)	0.45–4.50	0.95	3.19	2.99	0.57	**<** **0.01**

TFTs over the preceding 2 years showed elevated fT4 with unsuppressed TSH, consistent with inhibition of 5'-deiodinase with decreased deiodination of T4 to triiodothyronine (T3) by amiodarone (3) (Table 1). The marked increase in fT4 over just 3 months is consistent with the well-described sudden and explosive onset of AIT (1, 4) (Table 1).

Apart from mild weight loss, there were no symptoms of thyrotoxicosis, neck swelling or visual symptoms. There was no prior personal or family history of thyroid disease. He was afebrile and hemodynamically stable (blood pressure 126/98 mmHg, pulse rate 83 beats per minute), which was surprising given the degree of biochemical thyrotoxicosis. This could be attributed to *in-situ* pacemaker with a set rate of 50 beats per minute, as well as chronic beta-blocker and amiodarone therapy. Significant findings included fine hand tremors and a small diffuse goiter, but no signs of thyroid eye disease or pretibial myxedema. There was central cyanosis, digital clubbing and a pan-systolic cardiac murmur. His pulse was regular and there were no signs of heart failure.

A provisional diagnosis of AIT was made. Empirical treatment for both subtypes was initiated with carbimazole 40 mg and prednisolone 40 mg daily, while awaiting further investigations to distinguish between AIT type 1 (AIT1) and type 2 (AIT2). Amiodarone was discontinued. Close liver function monitoring was ordered due to carbimazole use in the setting of hyperbilirubinemia (related to hepatic venous congestion). TSH-receptor antibodies and anti-TPO antibodies were negative, while ultrasonography showed thyroid nodules with patchy parenchymal flow. The initial report of the Technetium (^99^Tc) pertechnetate scan described diffusely increased thyroid uptake, which suggested AIT1 (5, 6). Thus, prednisolone was stopped while carbimazole was continued. However, subsequent development of neutropenia attributed to acute viral illness necessitated carbimazole cessation. Reintroduction of prednisolone at this juncture led to significant reduction in serum thyroxine, but this also had to be discontinued within 1 week due to worsening hepatic dysfunction from acute hepatitis E infection (Table 2).

**Table 2:** Summary of clinical course with corresponding treatment.

**Investigations**	**Reference interval**	**Number of weeks after diagnosis of amiodarone induced thyrotoxicosis**										
		**0**	**2**	**3**	**4**	**6**	**7**	**11**	**12**	**15**	**23**	**30**
fT4 (pmol/L)	8.0–16.0	**>** **72.0**	**>** **72.0**	**68.6**	**70.9**	**67.8**	**51.7**	**47.2**	**38.5**	**18.0**	**5.4**	16.0
TSH (mIU/L)	0.45–4.50	**<** **0.01**	**<** **0.01**	**<** **0.01**	**<** **0.01**	**<** **0.01**	**<** **0.01**	**<** **0.01**	**<** **0.01**	**0.14**	**41.39**	2.66
Free T3 (pmol/L)	3.5–6.0			**11.3**								
Total T3 (nmol/L)	0.90–2.60					2.14	1.15					
**Medications**												
Carbimazole		**√**	**√**	**√**	**√**							
Thiamazole								**√**	**√**	**√**	**√**	
Bisoprolol			**√**	**√**	**√**	**√**	**√**	**√**	**√**	**√**	**√**	**√**
Propranolol						**√**	**√**	**√**	**√**	**√**		
Prednisolone		**√**				**√**						
Cholestyramine				**√**	**√**	**√**	**√**	**√**	**√**			
Lithium							**√**	**√**	**√**			
Sequence of events			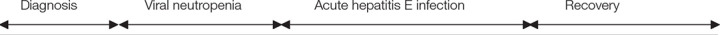									

The option of total thyroidectomy was considered but abandoned due to prohibitively high surgical and anesthetic risk. Radioactive iodine (RAI) ablation was unlikely to be successful due to low radioactive iodine uptake (<2%) after low dose (100 micro-CI) I-131 thyroid scan. This was discrepant from the ^99^Tc scan; a false negative result was unlikely with no recent exposure to iodinated contrast, and thionamides were held off before the scan (7). A possible reason for this discrepancy was that unlike RAI, pertechnetate is rapidly uptaken but not organified. However, when reviewed by another radiologist, interpretation of the ^99^Tc scan was that of decreased uptake – consistent with the I-131 scan.

Second-line agents – cholestyramine, beta-blockers and lithium, were required to decrease the risk of cardiovascular strain (Table 2). The lack of significant reduction in serum thyroxine despite cholestyramine prompted initiation of propranolol followed by lithium, which was added as a last resort due to its narrow therapeutic index. In our patient, lithium levels were monitored and there were no adverse effects.

Following improvement in hyperbilirubinemia, he was started on thiamazole, which does not require hepatic activation. When he had achieved near-euthyroid status, lithium and cholestyramine were stopped (Table 2). Throughout this entire period, our patient remained stable with no cardiovascular complications.

The patient developed hypothyroidism about 6 months after therapy initiation, and thiamazole was gradually tapered and stopped (Table 2). Post cessation of thiamazole, thyroid function remained normal; but he suffered a recurrence of atrial flutter and supraventricular tachycardia which was treated with nodal radiofrequency ablation. As there were no further arrhythmias, re-initiation of amiodarone was not required.

Throughout this entire journey, we ensured that our patient was closely updated and fully aware of his condition and progress. Before each treatment decision, the risks and benefits were explained in detail, so that an informed decision could be made. For example, before starting lithium, we explained that its utility was to reduce thyroid hormone levels in view of complications to other forms of treatment. However, the potential adverse effects include tremors, diarrhea, polyuria, polydipsia and hypercalcemia (8). Thus, there was a need for close follow up and monitoring of lithium levels. Our patient was adherent to all treatment and follow-up. Indeed, till present, he remains appreciative of our efforts.

## Discussion

AIT is a diagnostic dilemma. Typical clinical features of thyrotoxicosis may be absent because amiodarone leads to decreased catecholamine receptors, decreased T3 effect on beta-adrenoceptors and inhibition of T4 to T3 conversion (9), consistent with the observation in our patient. To compound this diagnostic difficulty, management of AIT carries high stakes. AIT is associated with increased morbidity and mortality, and all patients are considered at risk of emergency treatment (10).

Establishing the AIT subtype is often challenging but crucial in determining the optimal treatment strategy (11). Amiodarone is a benzofuranic antiarrythmic drug that contains ~37% iodine by weight (7). AIT1 is due to excess thyroid hormone synthesis from the high iodine content of amiodarone through the Jod-Basedow effect (3, 9). This usually occurs in patients with pre-existing thyroid disease such as latent Graves Disease or nodular goiter, although the role of amiodarone in triggering thyroid autoimmunity is not well established (1, 9). First-line treatment is thionamides (1, 12), although higher doses and longer treatment are often required due to increased intra-thyroidal iodine load (9). In contrast, AIT2, which usually occurs in normal thyroid glands, is caused by direct cytotoxic effects of amiodarone and/or its metabolite (desethylamiodarone) on thyroid follicular cells, leading to destructive thyroiditis (3). The ideal treatment is glucocorticoids, due to anti-inflammatory and membrane-stabilizing effects with decreased T4 to T3 conversion (9). Mixed AIT is also recognized, in which both pathogenic mechanisms are implicated and features of both subtypes are often present (1, 7).

As our patient had multiple features which supported both AIT1 and AIT2 (Table 3), the AIT subtype was initially unclear, and the possibility of mixed AIT was considered. However, with the benefit of hindsight, the likely diagnosis was AIT2 rather than mixed AIT (Table 3). To begin with, AIT2 is more likely in Singapore, an iodine sufficient region. This diagnosis was supported by the absence of underlying thyroid disease, long duration of prior amiodarone exposure, negative thyroid antibodies and decreased thyroid scan uptake. Despite an apparent initial lack of response, the significant response to corticosteroid reintroduction observed is consistent with a delayed response to corticosteroids in AIT2 patients with markedly elevated thyroid hormone and large thyroid volumes (13), although this could only be given for 1 week due to contraindications. Together with the relatively short duration of thyrotoxicosis and maintenance of euthyroidism without need for further treatment, this further confirmed AIT2.

**Table 3:** Features of AIT Type 1 and 2 in our patient.

	**AIT Type 1**	**AIT Type 2**
Gender		+ (Male^9^)
Underlying thyroid disease		+ [No (1, 3, 9)]
Frequency		+ [Much more common (14)]
Geographical location		+ [Singapore is an iodine-sufficient region (15)]
Duration of amiodarone exposure prior to development of thyrotoxicosis		+ [32 months of preceding amiodarone therapy (16)]
Thyroid hormone levels		+ [Higher (17)]
Thyroid autoantibodies		+ [Absent (18)]
Thyroid ultrasound	+ [Mildly enlarged thyroid lobes with nodules up to 1 cm (5, 6)]	
Color flow Doppler	+ [Patchy parenchymal flow (6)]	
Tc-99 m thyroid scan		+ [Decreased uptake (5)]
I-131 thyroid scan		+ [Decreased uptake (6)]
Duration of hyperthyroidism		+ [Euthyroid within 3–5 months of amiodarone withdrawal (3)]
Response to treatment	+ [No reduction in fT4 levels despite 2 weeks of combined treatment with thionamide and high dose prednisolone^7^]	+ [Subsequent marked reduction in fT4 levels with reintroduction of corticosteroids (19)]+ [No significant response to 5 weeks of high-dose thionamides]
Spontaneous remission		+ [Developed euthyroidism without need for any definitive or maintenance anti-thyroid treatment (1)]
Require subsequent therapy for thyroid disease		

As outlined above, significant difficulties in management were encountered. This patient developed neutropenia which limited high dose thionamide use, which was not surprising as the risk of carbimazole-induced agranulocytosis is dose-dependent and usually occurs in the first 100 days of therapy (7); as well as acute hepatitis E infection, which necessitated corticosteroid cessation. At the same time, due to severe biochemical thyrotoxicosis and underlying cyanotic congenital heart disease, urgent reduction of serum thyroxine was essential to decrease the risk of cardiovascular decompensation (7). Thus, thyroidectomy was an important consideration in this patient. In a Mayo clinic case series of 17 AIT patients who underwent thyroidectomy, the indication was severe thyrotoxicosis requiring prompt resolution and intolerance to anti-thyroid drugs in 35 and 24% of patients, respectively (20); with both of these indications applicable in this patient. Similarly, in another case series, the indication for thyroidectomy in 27% of patients was side-effects of anti-thyroid drugs (21). The main effect of lithium is inhibition of T4 release (22), while cholestyramine increases enterohepatic clearance of thyroid hormone (23). Although lithium and cholestyramine can be used to treat thyrotoxicosis, reported successful use of these agents in AIT is limited. Lithium has been shown to decrease the time required to achieve euthyroidism in AIT, although not specifically in AIT2 (22). In a case series of 5 AIT2 patients, 2 of them received both cholestyramine and lithium but eventually still required thyroidectomy (24). In our patient, lithium and cholestyramine were crucial in restoring euthyroidism and successfully averting high-risk thyroidectomy.

On top of this, an additional difficulty was the controversial initial thyroid uptake scan report of increased tracer uptake which suggested AIT1, leading to corticosteroids being prematurely stopped after 2 weeks. That said, this was unlikely to have a significant overall impact on management due to subsequent contraindication to corticosteroids.

An important issue we considered was continuation or cessation of amiodarone. Amiodarone is an efficient drug for life-threatening arrythmias, has a long half-life of 90 days and a cardioprotective effect (9, 10, 25). Thus, stopping amiodarone might increase the risk of life-threatening arrythmias without any short-term benefit (9, 25). Although amiodarone continuation might be feasible in AIT2 [(26, 27), p. 2], this might lead to increased recurrence rate of thyrotoxicosis and deleterious cardiac effects (28). In our patient, as underlying atrial flutter and heart block had been treated and initial AIT subtype was unclear, we chose to discontinue amiodarone with close monitoring for recurrence of arrythmia.

## Conclusion

AIT is often an insidious condition which is nonetheless important to urgently recognize and treat. In clinical practice, subtype determination is often challenging and might only be possible retrospectively, limiting the application of subtype-driven treatment. Physicians should recognize the need for urgent empirical treatment in patients with cardiac disease and establish close monitoring for complications of high-dose thionamides and corticosteroids. Where there are complications to first-line treatment, the expedient use of alternative treatment options is essential. This was observed in this case – successful treatment of AIT2 with lithium and cholestyramine.

## Data Availability Statement

The original contributions presented in the study are included in the article/supplementary material, further inquiries can be directed to the corresponding author/s.

## Ethics Statement

Written informed consent was obtained from the individual(s) for the publication of any potentially identifiable images or data included in this article.

## Author Contributions

MC and SM made substantial contributions to the conception and design of this manuscript. MC and SM worked on the initial drafts and subsequently revised it critically for important intellectual content. MC and SM have viewed and are in approval of the final version of the manuscript to be published. MC and SM agree to be accountable for all aspects of the work. All authors contributed to the article and approved the submitted version.

## Conflict of Interest

The authors declare that the research was conducted in the absence of any commercial or financial relationships that could be construed as a potential conflict of interest.
